# P-1922. Liver Function Test Abnormalities near Admission Predict Mortality for Patients with COVID-19: A Single-Center Study

**DOI:** 10.1093/ofid/ofae631.2082

**Published:** 2025-01-29

**Authors:** Lucia Cabrejos Hirashima, Nicole Naiman, Mamta K Jain

**Affiliations:** University of Texas Southwestern Medical Center, Dallas, Texas; UT Southwestern Medical Center, Dallas, Texas; UT Southwestern Medical Center, Dallas, Texas

## Abstract

**Background:**

Early data from the COVID-19 pandemic shows patients commonly present with abnormal liver function tests (LFTs) upon admission. This phenomenon is multifactorial and is associated with increased in-hospital mortality and disease severity. There is limited data describing COVID-19 LFT derangements in the United States, and few comparisons of LFT derangements between variants.

**Figure:**
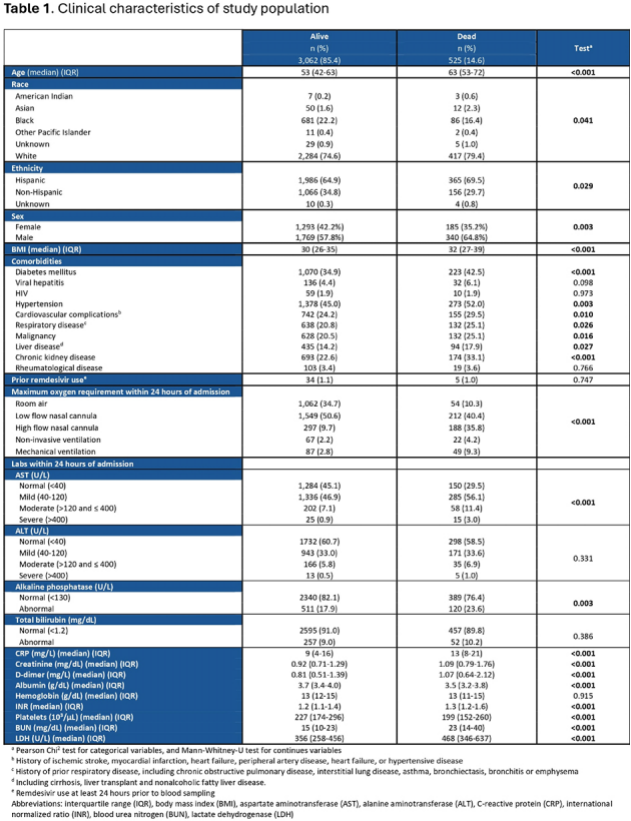

**Methods:**

This is a retrospective cohort study that includes non-pregnant adults admitted at Parkland Health and Hospital System from 03/01/2020 to 12/31/2021 with laboratory confirmed or ICD-10-documented COVID-19. We examine the association of LFT derangement (within 24 hours of admission) and mortality using multivariate logistic regression adjusting for baseline demographics, comorbidities, oxygen requirement, and lab values previously reported to be associated with COVID-19 mortality. We also examine this association after excluding patients with prior liver disease, viral hepatitis, and use of remdesivir prior to measuring LFTs. Finally, we compare LFT derangement patterns between variants (defined by date).

**Table 2.**
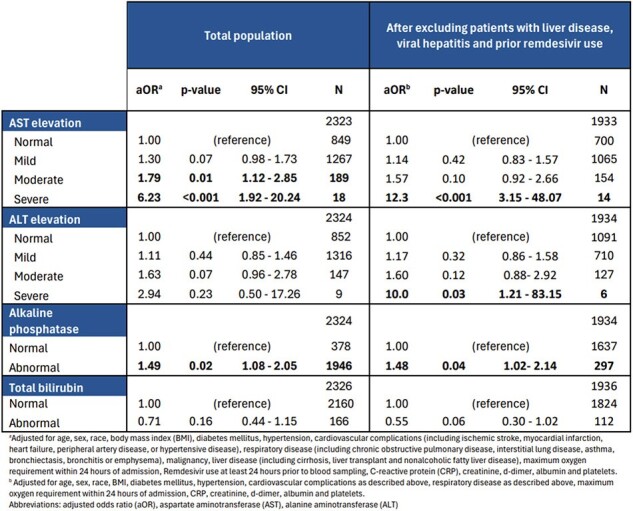
Multivariate logistic regression of liver function tests as predictors for mortality

**Results:**

In our cohort of 3587 patients, over 93% had an available LFT. Abnormalities of aspartate aminotransferase (AST) and alkaline phosphatase (AP) are more prevalent in the deceased group (Table 1). Multivariate logistic regression shows that severely elevated AST and abnormal AP are consistently associated with mortality, while severely elevated alanine aminotransferase (ALT) is associated with mortality only in patients with no prior liver disease, viral hepatitis or remdesivir use (Table 2). Our analysis reveals significant differences in the frequency of AST and ALT abnormalities based on the variant, with the delta variant showing the highest occurrence of abnormalities. However, we do not find a significant difference in mortality between variants (Table 3).

**Table 3.**
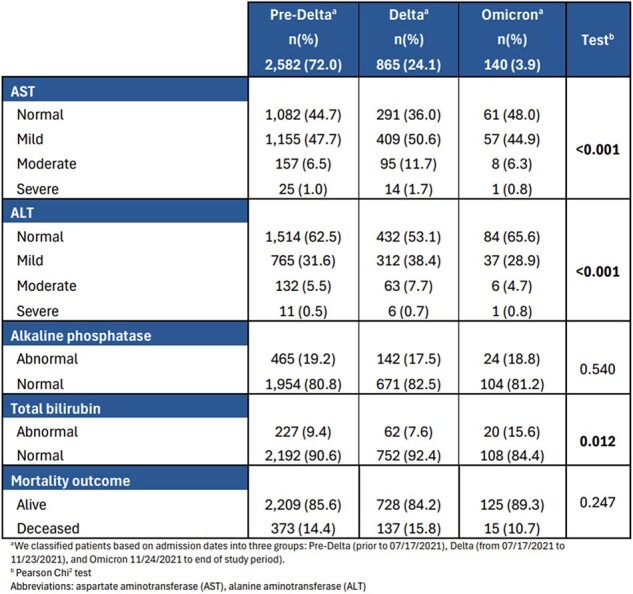
Distribution of liver function tests and mortality rate by SARS-CoV-2 variant

**Conclusion:**

This study highlights the consistent association between elevated LFTs at admission and mortality in COVID-19 patients, suggesting close monitoring by clinicians. It also demonstrates that COVID-19 is a systemic illness. Further studies are warranted to elucidate variant-specific differences in the association between LFT derangements and mortality.

**Disclosures:**

Mamta K. Jain, MD, MPH, Abbvie: Grant/Research Support|Gilead Sciences: Grant/Research Support|Laurent: Grant/Research Support

